# High Light Induced Disassembly of Photosystem II Supercomplexes in Arabidopsis Requires STN7-Dependent Phosphorylation of CP29

**DOI:** 10.1371/journal.pone.0024565

**Published:** 2011-09-07

**Authors:** Rikard Fristedt, Alexander V. Vener

**Affiliations:** Division of Cell Biology, Department of Clinical and Experimental Medicine, Linköping University, Linköping, Sweden; United States Department of Agriculture, Agricultural Research Service, United States of America

## Abstract

Photosynthetic oxidation of water and production of oxygen by photosystem II (PSII) in thylakoid membranes of plant chloroplasts is highly affected by changes in light intensities. To minimize damage imposed by excessive sunlight and sustain the photosynthetic activity PSII, organized in supercomplexes with its light harvesting antenna, undergoes conformational changes, disassembly and repair via not clearly understood mechanisms. We characterized the phosphoproteome of the thylakoid membranes from *Arabidopsis thaliana* wild type, *stn7*, *stn8* and *stn7stn8* mutant plants exposed to high light. The high light treatment of the wild type and *stn8* caused specific increase in phosphorylation of Lhcb4.1 and Lhcb4.2 isoforms of the PSII linker protein CP29 at five different threonine residues. Phosphorylation of CP29 at four of these residues was not found in *stn7* and *stn7stn8* plants lacking the STN7 protein kinase. Blue native gel electrophoresis followed by immunological and mass spectrometric analyses of the membrane protein complexes revealed that the high light treatment of the wild type caused redistribution of CP29 from PSII supercomplexes to PSII dimers and monomers. A similar high-light-induced disassembly of the PSII supercomplexes occurred in *stn8*, but not in *stn7* and *stn7stn8*. Transfer of the high-light-treated wild type plants to normal light relocated CP29 back to PSII supercomplexes. We postulate that disassembly of PSII supercomplexes in plants exposed to high light involves STN7-kinase-dependent phosphorylation of the linker protein CP29. Disruption of this adaptive mechanism can explain dramatically retarded growth of the *stn7* and *stn7stn8* mutants under fluctuating normal/high light conditions, as previously reported.

## Introduction

The light energy utilization in plant photosynthesis is regulated in response to ever changing environmental light intensities. This proceeds in photosynthetic membranes, called thylakoids, which are densely folded inside chloroplasts and heavily packed with protein-pigment complexes [1], [2], [3]. The highly stacked membrane layers are enriched in photosystem II (PSII) which uses the light energy to extract electrons from water and produce oxygen [4]. PSII is organized into large supercomplexes with variable amounts of peripheral light harvesting complexes (LHCII) [5] consisting of *lhcb1*, *lhcb2* and *lhcb3* gene products [6]. This outer antenna is connected to PSII via the minor CP29, CP26 and CP24 antenna proteins encoded by *lhcb4*, *lhcb5* and *lhcb6* genes, respectively [5], [7], [8]. PSII supercomplexes are dimeric and contain from two to four copies of trimeric LHCII complexes, with a further tendency to associate into megacomplexes, of which several types have been characterized [5]. The composition of the PSII supercomplexes changes depending on light conditions. Under low light a mobile part of the LHCII complex can migrant from PSII to photosystem I (PSI) [9], [10], [11] balancing the excitation energy between the photosystems in the process of state transitions [12]. The excessive light causes photoinactivation of oxygen-evolving PSII and significant decrease in the photosynthetic efficiency [13]. To deal with this problem the excess energy is released as a heat in the process known as non-photochemical quenching, which also involves reorganization and redistribution of PSII and its antenna complexes within the membranes [14], [15]. Additionally, the high light causes damage to the PSII protein pigment complex, in particular to the D1 reaction centre protein, which requires stepwise disassembly of PSII and its repair to sustain the photosynthetic function [13].

The recent years had witnessed discoveries demonstrating that environmentally-dependent differential phosphorylation of thylakoid membrane proteins regulates lateral migration [16], [17], mobility and packing density [18], composition [10], [11], stability and repair [19], [20] of the membrane protein complexes, as well as the whole macroscopic structure of thylakoids [20], [21]. These findings became possible after identification of the protein kinases responsible for phosphorylation of the thylakoid proteins [16], [17]. In the model plant Arabidopsis these thylakoid associated Ser-Thr kinases are called STN7 and STN8. STN7 is required for phosphorylation of LHCII polypeptides and TSP9, a soluble protein involved in regulation of light harvesting [12], [16], [17], [22], [23]. The phosphorylation of PSII core proteins is mediated through the STN8 kinase [17], which is essential for light-dependent phosphorylation of the D1, D2, CP43 and PsbH proteins of PSII, and of the calcium-sensing receptor (CaS) protein [24], [25]. The high level of PSII phosphorylation in plants adjusts macroscopic folding of thylakoid membranes: Arabidopsis mutants lacking STN8 have the membrane stacks that are markedly bigger than in thylakoids of wild type plants [20]. This increased membrane stacking obstructs lateral migration of membrane proteins, and thus suppresses turnover of damaged D1 in the plants exposed to high light [19], [20]. The loss of STN7 does not affect thylakoid stacking [20], however, it blocks state transitions and migration of mobile LHCII from PSII to PSI [12], [17]. In plants deficient in the protein phosphatase PPH1/TAP38, which is largely responsible for dephosphorylation of Lhcb1 and Lhcb2 proteins, the state transitions are blocked in the state of increased antenna size of PSI [26], [27]. Thus, reversible LHCII phosphorylation controlled by STN7 and PPH1/TAP38 is essential for state transitions in plants, while additional phosphorylation of the CP29 protein is required for state transitions in green algae *Chlamydomonas reinhardtii* [28].

The photosynthetic state 1 to state 2 transition in *Chlamydomonas reinhardtii* involves a functional coupling of phosphorylated CP29 protein to PSI [10], [11]. Exposure of the alga cells to photosynthetic state 1 or state 2 causes phosphorylation of CP29 at either two or four different sites, respectively, while high light treatment leads to its hyperphosphorylation at seven distinct residues [29]. The latter hyperphosphorylation was suggested to control uncoupling of light harvesting proteins from PSII under high light [30]. In Arabidopsis there are three isoforms of CP29: Lhcb4.1, Lhcb4.2 and Lhcb4.3; with Lhcb4.1 and Lhcb4.2 being the major expressed variants [31]. A large-scale Arabidopsis phosphoproteome profiling revealed phosphorylation of Lhcb4.1 at Thr^72^ or Thr^74^ and phosphorylation of Lhcb4.2 at Thr^78^ or Thr^80^ [32], while dependence of these modifications on light conditions or on particular protein kinases had not been studied. Only the N-terminal phosphorylation of Lhcb4.2 at Thr^6^ is known as STN7-dependent [12]. STN7 is required for phosphorylation of the LHCII polypeptides, which occurs under low light, but is deactivated under high light [12], [17], [33], [34], [35]. In this respect it is difficult to explain why growth of the *stn7* and *stn7stn8* mutants lacking STN7 is severely retarded under fluctuating high/low light conditions in comparison with *stn8* and wild type plants [33]. Moreover, no detailed comparative analysis of thylakoid protein phosphorylation in Arabidopsis wild type, *stn7*, *stn8* and *stn7stn8* plants exposed to high light had yet been reported.

In this work we analyzed the phosphoproteome of the thylakoid membranes in Arabidopsis wild type, *stn7*, *stn8* and *stn7stn8* plants exposed to high light. We mapped the sites of phosphorylation in the membrane proteins, quantified phosphorylation of the PSII core proteins under high light and found high-light- and STN7-dependent phosphorylation of CP29 variants Lhcb4.1 and Lhcb4.2. Using immunoblotting and mass spectrometry we conducted a study on the differences in composition of thylakoid protein complexes separated by blue native gel electrophoresis from the plants exposed to normal or high light. We revealed that high light treatment caused relocation of the CP29 protein from the PSII supercomplexes to PSII monomers and dimers and this movement, as well as the phosphorylation of CP29, was lost in the mutant plants lacking the STN7 protein kinase.

## Results and Discussion

### Phosphoproteome of thylakoid membranes in Arabidopsis exposed to high light

To characterize the phosphoproteome of thylakoid membranes in leaves of Arabidopsis plants exposed to high light we used mass spectrometric approach [30]. Thylakoid membranes were isolated from Arabidopsis plants exposed to either high light or control normal light and the surface-exposed phosphorylated protein domains were cleaved from the membranes by trypsin [36]. The phosphorylated peptides were then enriched by IMAC procedure [37], separated by nano-high performance liquid chromatography and identified by sequencing using an ion trap mass spectrometer performing alternating collision induced dissociation (CID) and electron transfer dissociation (ETD) of peptide ions [20]. The phosphorylated peptides presented in Table 1 were considered as identified in the samples from the high-light-treated leaves if their CID or ETD spectra had the MASCOT scores above 35 and expect values below 0.01 (Figure S1). All of these peptides but one were sequenced earlier from the plants exposed to normal light [12], [25], [32], [37]. Application of the novel ETD technique in the present study allowed identification of an additional peptide corresponding to triply phosphorylated N-terminus of the PSI protein PsaD (Table 1, Figure S1), while only a single phosphorylation of PsaD had been characterized before [37]. The major qualitative change caused by the high light treatment consisted in the well known [12], [17], [33], [34] dephosphorylation of the LHCII polypeptides (Table 1). To make a comprehensive analysis we also characterized phosphorylated peptides isolated from the leaves of the high-light-exposed T-DNA knockout mutants *stn7*, *stn8* and *stn7stn8*, deficient in the STN7 and STN8 kinases involved in phosphorylation of several major thylakoid proteins [12], [17], [20], [22], [24], [25]. Notably, phosphorylation of the PSI proteins PsaP and PsaD has not been affected in the *stn7*, *stn8* and *stn7stn8* mutants (Table 1), providing a strong evidence of the PSI phosphorylation by some other protein kinase. In agreement with this, acidic residues in the vicinity of the phosphorylated sites in PsaP and PsaD are characteristic for casein kinase II substrate recognition motifs [32]. This was confirmed by the online protein kinase motif prediction tools PhosMotifFinder and GPS 2.1, implying a possible role of chloroplast casein kinase II in the high light regulated phosphorylation responses. Moreover, we found for the first time that phosphorylation of the four amino acid residues in two isoforms of the minor antenna protein CP29, Lhcb4.1 (phosphorylation at Thr^72^ or Thr^74^) and Lhcb4.2 (phosphorylation at Thr^78^ or Thr^80^), was STN7-dependent (Table 1).

**Table 1:** Phosphorylated peptides identified by mass spectrometry from the leaves of wild type and *stn* mutant plants exposed to normal or high light.

Protein	Peptide sequence	Normal	High light
		wild type		*stn7*	*stn8*	*stn7stn8*
CP43	*Ac*-tLFNGTLALAGR	+	+	+	+	−
D2	*Ac*-tIALGK	+	+	+	+	+
D1	*Ac*-tAILER	+	+	+	+	−
P-PsbH	At^2^QTVEDSSR	+	+	+	+	+
PP-PsbH	At^2^Qt^4^VEDSSR	+	+	+	−	−
PsaP	At^65^TEVGEAPATTTEAETTELPEIVK	+	+	+	+	+
	ATt^66^EVGEAPATTTEAETTELPEIVK	+	+	+	+	+
CaS	SGt^380^KFLPSSD	+	+	+	−	−
PsaD	t^3^Ds^5^s^6^AAAAAAPATK	+	+	+	+	+
Lhcb2.1 Lhcb2.2 Lhcb2.4	*Ac*-RRt^3^VK	+	−	−	−	−
Lhcb1.1 Lhcb1.2 Lhcb1.3 Lhcb1.5	*Ac*-RKt^3^VAKPK	+	−	−	−	−
CP29 (Lhcb4.2)	FGFGt^6^KK	+	+	+	+	−
CP29 (Lhcb4.1)	NLAGDVIGt^72^RTEAADAK	+	+	−	+	−
	NLAGDVIGTRt^74^EAADAK	+	+	−	+	−
CP29 (Lhcb4.2)	NLYGEVIGt^78^RTEAVDPK	+	+	−	+	−
	NLYGEVIGTRt^80^EAVDPK	+	+	−	+	−

The modifications in peptide sequences are indicated like: *Ac*-, N-terminal acetylation; low case t, phosphorylated threonine residue; low case s, phosphorylated serine residue, with superscripts indicating the numbers of phosphorylated residues in the protein sequence. All of these phosphorylated peptides, with exception of the triply phosphorylated peptide from the PsaD protein, had been sequenced earlier from the plants exposed to normal light [12], [25], [32], [37]. The phosphorylated peptides were considered as identified in the samples from the high-light-treated plants on the basis of CID or ETD sequencing data presented in Figure S1.

### Quantification of PSII phosphorylation in Arabidopsis plants exposed to high light

**Figure 1:**
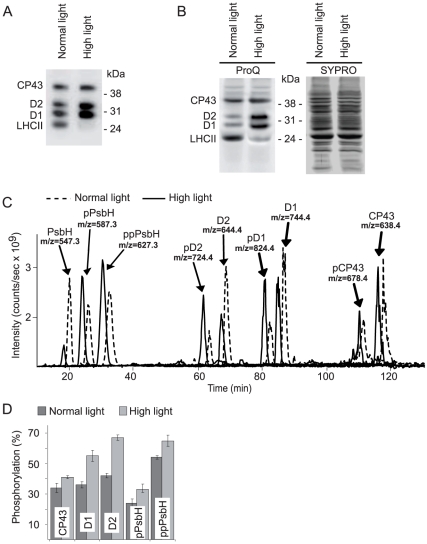
Phosphorylation of the PSII core proteins in plants exposed to normal or high light. **A**) Immunoblotting analysis of SDS-PAGE separated thylakoid proteins using anti-phosphothreonine antibody. Thylakoids were isolated from wild type plants exposed for three hours to either normal light (120 µmol photons m^−2^ s^−1^) or high light (900 µmol photons m^−2^ s^−1^), as indicated. **B**) ProQ Diamond phosphoprotein stain and Sypro Ruby total protein stain, as indicated, of the same gel with SDS-PAGE separated thylakoid proteins from the plants exposed to either normal light or high light, like in A. The phosphoproteins detected in A and B are labeled on the basis of the previous studies [12], [20], [24], [35]. **C**) LC-MS extracted ion chromatograms of the phosphorylated and non-phosphorylated N-terminal peptides of the PSII core proteins from plants exposed to normal light (dashed line) or high light (solid line). The peptide ion peaks are labeled with corresponding protein name and mass over charge ratio (m/z). The peptide retention times were similar in separate LC-MS runs and the dashed line is shifted to the right from the solid line artificially to allow clear comparison of the peak intensities in two chromatograms. **D**) The level of phosphorylation for each of the PSII core proteins from plants exposed to normal or high light, calculated from the corresponding ratios of phosphorylated to non-phosphorylated peptide intensities of the LC-MS profiles like in C using the earlier determined flyability ratios for peptide/phosphopeptide pairs from the D1, D2, CP43 and PbsH proteins [21]. The values are means ± SE of four experiments in each condition.

The high light treatment of plants increased phosphorylation of the PSII core proteins, as clearly visualized either by immunoblotting with the antibody against phosphothreonine (Figure 1A) or by Pro-Q Diamond phosphoprotein gel stain (Figure 1B). To quantify the increase in PSII phosphorylation we used mass spectrometry. Thylakoids isolated from plants adapted to either normal or high light were treated with trypsin to cleave the surface-exposed phosphorylated and non-phosphorylated parts of the membrane proteins, which were then separated and quantified using liquid chromatography and mass spectrometry (LC-MS). The major phosphorylated peptides from PSII proteins and their non-phosphorylated counterparts were successfully resolved and the intensities of their signals were determined (Figure 1C). To measure the absolute phosphorylation stoichiometry [38] we used a normalization procedure that accounted for the differences in ionization and in signal intensities of phosphorylated and corresponding non-phosphorylated peptides [21], [38]. This was done using the earlier determined flyability ratios for peptide/phosphopeptide pairs from the D1, D2, CP43 and PbsH proteins [21]. The ratios of the normalized signals for each phosphopeptide/peptide pair determined the extent of phosphorylation for the PSII proteins (Figure 1D). The similar quantitative measurements had earlier been done only for plants adapted to either normal light or darkness [21]. Exposure of the plants to high light increased phosphorylation of D1 and D2 by 20% and that of CP43, as well as singly and doubly phosphorylated PsbH by about 10%. The quantitative data (Figure 1D) and account of the equivalent molar amounts of the CP43, D1, D2 and PsbH proteins in the PSII core allowed calculation of average 2.4±0.1 or 3.1±0.2 phosphoryl groups per PSII monomer in the plants treated by either normal or high light, respectively. The PSII phosphorylation is required to support turnover of damaged PSII reaction centre protein D1 in the leaves exposed to high light and it depends on the STN8 protein kinase [18], [19], [20], [21]. It is most likely that the increase in PSII phosphorylation induced in plants under high light enhances the mobility of proteins within the thylakoid membrane and consequently turns the membranes into a more dynamic state [18].

### Phosphorylation of the CP29 protein in the plants exposed to high light

**Figure 2:**
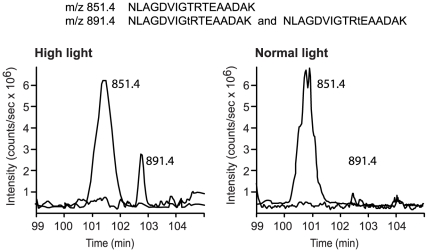
Increase in phosphorylation of Lhcb4.1 protein after expose of plants to high light. LC-MS extracted ion chromatograms of the phosphorylated and non-phosphorylated Lhcb4.1 peptides from plants exposed to high light or normal light, as indicated. Chromatograms for the ion with mass over charge ratio (m/z) 851.4 correspond to the non-phosphorylated Lhcb4.1 peptide with the shown sequence. Chromatograms for the ion with mass over charge ratio (m/z) 891.4 correspond to phosphorylated Lhcb4.1 peptides with the same mass but alternative phosphorylation of the threonine residues indicated in the sequences by low case t.

Analyzing the complex mixtures containing over a thousand peptides from thylakoids treated with trypsin we were also able to make quantitative measurements of phosphorylated and non-phosphorylated peptides from the Lhcb4.1 isoform of CP29 (Figure 2). In the plants treated with high light the relative phosphorylation level for this peptide was 19±8% (mean ± SE of five experiments). Phosphorylation of the same peptide from Lhcb4.1 was less than 3% when the plants were treated with normal light (Figure 2). These measurements were made without normalization for the difference in ionization of phosphorylated and non-phosphorylated peptides. Nevertheless, they demonstrated quantitative increase in phosphorylation stoichiometry of Lhcb4.1 upon the transfer of plants from normal to high light. The peptides from the CP29 protein (Figure 2) had a hundred times lower signal intensities than the peptides from the PSII core proteins (Figure 1C). That was mainly because of suppression of peptide signals from two isoforms of the CP29 protein, Lhcb4.1 and Lhcb4.2, by more abundant thylakoid peptides. Due to the same reason neither phosphorylated peptides from Lhcb4.2 (Table 1) nor their non-phosphorylated counterparts were detected during LC-MS analyses of the total thylakoid peptide mixtures. Thus, we made a relative quantification of the Lhcb4.2 phosphorylation level using a technique of stable isotope labeling, as described below.

**Figure 3:**
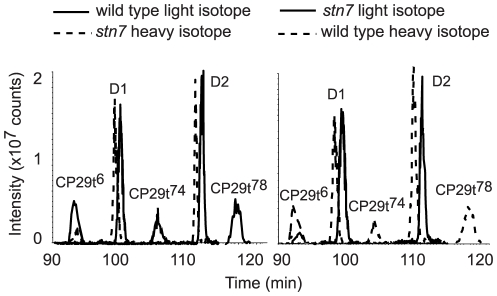
Comparative analysis of CP29 phosphorylation in the high light treated wild type and *stn7* mutant plants using stable isotope labeling and LC-MS. Extracted ion chromatograms of the phosphorylated peptides from CP29, with the superscript numbers indicating phosphorylated threonine residue according to Table 1, and from D1 and D2 proteins, as marked. The left chromatogram shows intensities of phosphorylated peptide signals from the wild type labeled with light isotope (solid line) and from *stn7* labeled with heavy isotope (dashed line). The right chromatogram shows the results of reciprocal labeling: the wild type labeled with heavy isotope (dashed line) and *stn7* labeled with light isotope (solid line). There was no significant difference in the retention times of the labeled peptides and the dashed lines are shifted to the left from the solid lines artificially to allow clear comparison of the peak intensities.

We found that high-light-induced phosphorylation of Lhcb4.1 and Lhcb4.2 at four different sites was STN7-dependent (Table 1). This was surprising because STN7 is essential for phosphorylation of the LHCII polypeptides, known to be deactivated under high light [12], [17], [33], [34]. The opposite effects of the high light treatment on STN7-dependent phosphorylation of either LHCII or CP29 are hard to explain without suggestion of different signaling chains [23] involved in phosphorylation of these proteins. The N-terminal phosphorylation of Lhcb4.2 at Thr^6^ has been detected in the *stn7* mutant (Table 1); however, it was difficult to determine if it was lower than in the wild type plants. To quantify differences between the high-light-induced phosphorylation of CP29 in *stn7* and the wild type plants we applied the technique of stable isotope labeling. The peptide mixtures released by trypsin from the wild type and mutant thylakoids were differentially labeled by hydrogen or deuterium containing methanol, mixed in 1∶1 ratio and enriched for the phosphorylated peptides by IMAC [24], [39]. The ratios of the heavy to light isotope labeled phosphorylated peptides were then determined using mass spectrometry [26]. The control experiments were also done with reciprocal isotope labeling of the wild type and mutant peptides. The extracted ion chromatograms for the differentially labeled phosphorylated peptides from CP29, as well from the D1 and D2 proteins, used like controls, are presented in Figure 3. Phosphorylated peptides from D1 and D2 were detected in the equal amounts, while phosphorylation of Lhcb4.1 at Thr^72^ or Thr^74^ and phosphorylation of Lhcb4.2 at Thr^78^ or Thr^80^ was found only in the wild type in both forward and reciprocal labeling experiments (Figure 3). We also found a distinct difference between the *stn7* mutant and wild type in the phosphorylation of CP29 (Lhcb4.2) at Thr^6^. This phosphorylation of CP29 in *stn7* was 4 to 5 times lower than in the wild type (Figure 3). Phosphorylation of Lhcb4.2 at Thr^6^ present in the *stn7* mutant indicated that several protein kinases are involved in phosphorylation of CP29, like it also occurs in green alga *Chlamydomonas reinhardtii* [40]. In total phosphorylation of two CP29 isoforms at five different threonine residues was either abolished or significantly reduced in the high-light-treated leaves of the *stn7* plants.

### High light induced relocation of CP29 protein from PSII supercomplexes

**Figure 4:**
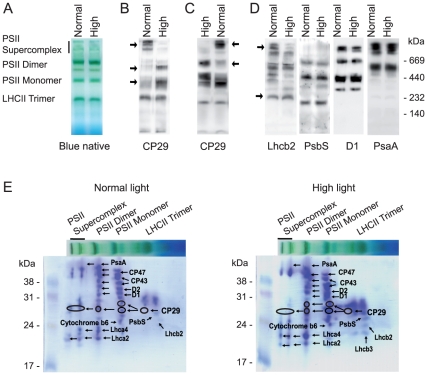
Blue native gel separation and analyses of the thylakoid membrane complexes from plants exposed for three hours to either normal or high light, as indicated. **A**) Representative blue native gels showing different PSII complexes. The samples loaded onto each gel lane contained the same amount of chlorophyll (8 µg). **B**) Immunoblotting of the blue native gels with the samples from the plants exposed to normal or high light using antibody against CP29. The arrows point towards the gel zones with significant changes in the amount of CP29. **C**) Immunoblotting of the blue native gels with antibody against CP29. The samples were from the plants exposed for three hours to high light and then transferred to normal light for 30 min, as indicated. The arrows point towards the gel zones with significant changes in the amount of CP29. **D**) Immunoblotting of the gels like in A using antibodies against the Lhcb2, PsbS, D1 and PsaA proteins, as indicated. The arrows point towards the gel zones with significant changes in the amount of Lhcb2. **E**) Two-dimensional gel analysis of thylakoid protein complexes from normal or high light treated plants, as indicated. Thylakoids were solubilized and separated by blue native electrophoresis (the horizontal lanes) and the gel lanes were subjected to denaturing SDS-PAGE in the second, vertical dimension. The gels were stained with Coomassie blue and the indicated spots were subjected to in-gel digestion and protein identification by LC-MS.

To search for the functional implications of the high-light-induced phosphorylation of CP29 we analyzed the distribution of this protein between different photosynthetic protein-pigment complexes. To this end we performed analyses of blue native gels containing separated thylakoid membrane complexes from plants exposed for 3 hours to either normal or high light (Figure 4A). Immunoblotting with antibody against CP29 detected most of this protein in the gel zones corresponding to PSII supercomplexes and LHCII trimers under normal light (Figure 4B). However, the high light caused relocation of CP29 from the PSII supercomplexes to the PSII dimers and monomers (Figure 4B). This migration was reversible: if the high-light-treated plants were transferred to normal light for 30 minutes CP29 relocated back to PSII supercomplexes (Figure 4C). The antibodies against a major light harvesting protein Lhcb2 and against the D1 protein of PSII also revealed reduced amounts of these proteins in the supercomplexes after the high light treatment (Figure 4D). These results are consistent with the recent investigation that used sucrose gradient fractionations of mildly solubilized thylakoids membranes and demonstrated fast high-light-dependent movement of CP29 and LHCII from the PSII supercomplexes [15]. An antibody against the PsbS protein detected PsbS associated mainly with PSII dimers, PSII monomers and LHCII trimers, and this localization did not change depending on the light intensity (Figure 4D). The PSI protein PsaA was also virtually immobile during the change in the light treatment (Figure 4D).

The complexes of PSII and PSI do not separate from each other during blue native electrophoresis (Figure 4D) [41], and reduction in the amount of the PSII supercomplexes is not clearly visible directly on the blue native gels after a relatively short exposure of plants to high light [19]. To verify the pronounced high-light-induced depletion of CP29 from the PSII supercomplexes observed with immunoblotting (Figure 4B), we employed 2D gel protein analysis of the thylakoid membrane samples from normal and high light treated plants. We cut all major protein spots from the 2D gel regions corresponding to the PSII supercomplexes, PSII dimers, PSII monomers and LHCII trimers and analyzed them by mass spectrometry [22]. The positions of several selected reference proteins in these gel regions are indicated in the Figure 4E. We specifically focused on the detection of CP29 in each of the analyzed gel regions in the samples from normal and high light treated plants. Notably, the samples from plants illuminated by high light revealed significant decrease in CP29 association with the PSII supercomplexes (Figure 4E). We measured intensities of the CP29-containing gel spots stained with Coomassie blue and calculated relative distribution of this protein in each 2D gel. In the samples from plants illuminated by normal light the relative distribution of CP29 between the PSII supercomplex, PSII dimer, PSII monomer and LHCII trimer was: 38±19%, 19±13%, 30±19% and 13±5%, respectively (mean ± SE from three 2D gels). In the samples from plants treated by high light the relative distribution of CP29 between the PSII supercomplex, PSII dimer, PSII monomer and LHCII trimer was: 14±12%, 32±11%, 42±13% and 12±11%, correspondingly (mean ± SE from three 2D gels). These results confirmed the immunological data on the CP29 depletion from PSII supercomplexes in the high light treated plants (Figure 4B).

Phosphorylation of CP29 from several species changes mobility of this protein during electrophoresis, which results in the appearance of two or more bands upon phosphorylation of CP29 at distinct residues [29]. The 2D gel protein analyses of thylakoid membrane samples detected two CP29 protein spots in the PSII monomers from either normal or high light treated plants (Figure 4E). The high light treatment resulted in appearance of a second CP29 protein spot in the PSII dimer (Figure 4E), which suggested a possible phosphorylation-dependent relocation of CP29 from PSII supercomplexes to PSII dimers.

**Figure 5:**
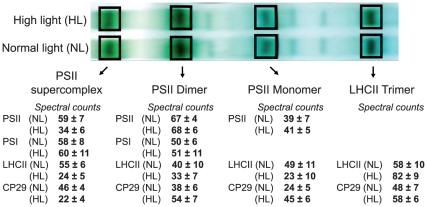
Blue native gel separation of the thylakoid membrane complexes from plants exposed to either normal or high light, as indicated, and their analyses using MS/MS spectral counts. The gel bands corresponding to PSII supercomplex, PSII dimer, PSII monomer and LHCII trimer were subjected to in-gel digestion and protein analyses by LC-MS. The numbers below the analyzed gel bands correspond to the sums of tandem mass spectra of two core proteins from each of PSII, PSI or LHCII (see Figure S2), as well as a sum of the spectral counts for the CP29 protein isoforms Lhcb4.1 and Lhcb4.2. The values are means ± SE of four independent experiments at each light condition.

To get additional quantitative insight into the high-light-induced redistribution of CP29 between the different membrane protein complexes separated by blue native electrophoresis we cut the corresponding gel bands containing multiple proteins, made in-gel digestion and analysis using nano-liquid chromatography and tandem mass spectrometry with the following spectral counting for proteins of interest. This method has been proven to provide an accurate correlation between the numbers of spectral counts acquired for each protein and a relative abundance of this protein in the analyzed mixture [31], [42]. We counted tandem mass spectra sums for the both CP29 isoforms Lhcb4.1 and Lhcb4.2 in the gel bands corresponding to PSII supercomplex, PSII dimer, PSII monomer and LHCII trimer. A similar analysis of the spectral counts has also been done for two core proteins from each of the three complexes: PSII (D1 and D2 proteins), PSI (PsaA and PsaB proteins) and LHCII (Lhcb1 and Lhcb2 proteins) (Figure S2 and Figure 5). These data revealed that transfer of plants from the normal to high light caused more than 50% decrease in CP29 association with the PSII supercomplexes (Figure 5), which was in a good agreement with the results obtained by either immunoblotting with the antibody against CP29 (Figure 4B) or 2D gel protein analysis (Figure 4E). The CP29 protein relocated from PSII supercomplexes mostly to PSII dimers and monomers, and to a lesser extent to LHCII trimers (Figures 4B, 4E and 5). The mobile part of LHCII, however, moved exclusively to LHCII trimers after the high light treatment (Figures 4D and 5). It is important to stress that the gel bands analyzed as the “LHCII trimer” were broad (Figure 5) and obviously contained the recently characterized B4C complex [15] composed of the monomeric Lhcb proteins CP29 and CP24, and the trimeric LHCII (see also Figure 4E). Dissociation of the B4C complex from PSII supercomplexes was implied in the initiation of non-photochemical fluorescence quenching and heat dissipation after exposure of plants to high light [15]. Our data demonstrate that high light induced disassembly of the PSII supercomplexes causes relocation of CP29 mostly to the PSII dimers and PSII monomers, and to a lesser extent to “LHCII trimers”/B4C complex.

The spectral count analysis (Figure 5) localized either 29±5% or 12±4% of the total CP29 in the PSII supercomplexes in the conditions of normal or high light, respectively. This corresponded to depletion of about 17% of the total CP29 from PSII supercomplexes upon the high light treatment. “The total CP29” was calculated as a sum of CP29 detected in the PSII supercomplexes, PSII dimers, PSII monomers and LHCII trimers from each blue native gel. The 2D gel protein analysis, described above, determined 38±19% of the total CP29 localized in the PSII supercomplexes of plants exposed to normal light. In the samples from plants treated by high light this number decreased to 14±12%, corresponding to depletion of about 24% of the total CP29 from PSII supercomplexes. Notably, relocation of 17 to 24% of the total CP29 from PSII supercomplexes upon the high light treatment, detected by two different techniques, correlated with about 19±8% increase in the phosphorylation of Lhcb4.1 (Figure 2). This correlation suggested that relocation of CP29 from PSII supercomplexes may be phosphorylation-dependent.

### Relocation of CP29 from PSII supercomplexes is STN7-dependent

**Figure 6:**
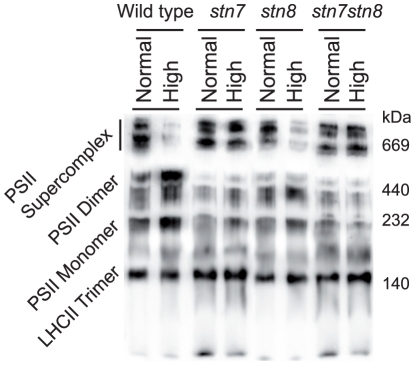
Immunoblotting of the blue native gels with thylakoid complexes from normal and high light treated wild type, *stn7*, *stn8* and *stn7stn8* plants, as indicated, using antibody against CP29.

To look for the mechanism behind the high-light-induced CP29 dissociation from the PSII supercomplexes we examined if this process is phosphorylation-dependent. Our finding that phosphorylation of Lhcb4.1 and Lhcb4.2 at four different sites was STN7-dependent (Table 1) suggested the analysis of the *stn7* and *stn7stn8* Arabidopsis mutants for the high-light-induced CP29 redistribution. We isolated thylakoids from these mutants, as well as from control wild type and *stn8* plants treated by either normal or high light and subjected them to solubilization and following blue native gel electrophoresis. In all plants exposed to normal light the distribution pattern of CP29 between different protein complexes looked very similar. However, after the high light treatment CP29 distribution between the thylakoid complexes was remarkably different between the STN7-deficient plants on the one hand and wild type and *stn8* on the other hand (Figure 6). The high light did not cause visible CP29 depletion from PSII supercomplexes in *stn7* and *stn7stn8*, according to the immunoblotting with CP29 specific antibody, whereas CP29 was barely detectable in the PSII supercomplexes of wild type and *stn8* after this light treatment (Figure 6). Immunoblots with D1, Lhcb1 and PsbS antibodies demonstrated that the distribution of these proteins did not differ significantly between the mutants, while the high light treatment caused more pronounced Lhcb1 depletion from the PSII supercomplexes in *stn8*, as compared with *stn7* and *stn7stn8* (Figure S3). These results demonstrate that deficiency in the STN7 protein kinase prevents the high-light-induced disassembly of the PSII supercomplexes in Arabidopsis, at least at the time scale of 3 hours.

### Conclusions

**Figure 7:**
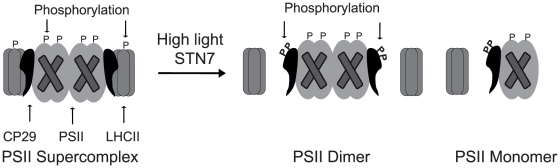
A model for the high-light- and STN7-dependent disassembly of PSII supercomplexes. The high light induced phosphorylation of CP29 causes disassociation of LHCII from PSII along the interface of the linker proteins, with the most of CP29 remaining connected with the PSII dimers and PSII monomers.

Analysis of the phosphoproteome in the thylakoid membranes from Arabidopsis wild type, *stn7*, *stn8* and *stn7stn8* plants exposed to high light revealed specific STN7-dependent multiple phosphorylation of the PSII linker protein CP29. Separation and characterization of the thylakoid protein complexes from the plants exposed to either normal or high light identified the high-light-dependent relocation of CP29 from the PSII supercomplex to PSII dimers and PSII monomers, as it schematically outlined in Figure 7. This relocation of CP29 was light-dependent and reversible in the wild type, but it did not occur in the *stn7* and *stn7stn8* plants. The latter plants differed from the wild type and *stn8* only in the absence of phosphorylation of Lhcb4.1 at Thr^72^ or Thr^74^ and of Lhcb4.2 at Thr^78^ or Thr^80^ upon transition from normal to high light. Thus, we postulate that disassembly of the PSII supercomplexes in plants exposed to high light operates via the STN7-kinase-dependent phosphorylation of Lhcb4.1 and Lhcb4.2 isoforms of the PSII linker protein CP29. Disruption of this adaptive mechanism can explain dramatically retarded growth of the *stn7* and *stn7stn8* mutants under fluctuating normal to high light conditions, as previously reported [33].

CP29 is the linker protein, which together with two other minor antenna proteins, CP26 and CP24, connects peripheral LHCII antenna to PSII, organizing large supercomplexes [5], [7], [8]. Under bright sunlight, when the amount of energy harvested by plants exceeds the electron transport capacity of PSII, CP29 participates in the charge-transfer quenching crucial to non-photochemical energy dissipation [43], [44], which also includes dissociation of the complex containing CP29, CP24 and LHCII from the PSII supercomplexes [15]. Our experimental data demonstrate that exposure of the *Arabidopsis thaliana* plants to high light causes multiple phosphorylation of CP29 and disassociation of LHCII from PSII along the interface of the linker proteins, with the most of CP29 remaining connected with the PSII dimers and PSII monomers (Figure 7). This molecular mechanism for potential protection of PSII from the excessive harvested light energy may be universal to land plants and can explain the earlier found requirement for the phosphorylation of CP29 in protecting maize from cold stress [45], winter rye from cold and high light stress [46], as well as barley from water [47] and other environmental stresses [48].

## Materials and Methods

### Plant Material

Arabidopsis thaliana wild type (ecotype Columbia) plants, *stn7* (SALK 073254) [17], *stn8* (SALK 060869) [24], and double mutant *stn7stn8* [17], [20] in Columbia background were grown hydroponically at 23°C, 65–70% relative humidity according to [49]. A photosynthetic flux of 120 µmol photons m^−2^ s^−1^ with a photoperiod of 8 h light and 16 h dark was used and in the case of high light experiments photosynthetic flux of 900 µmol photons m^−2^ s^−1^ was applied.

### Thylakoid isolation, Blue native PAGE, SDS-PAGE and Western blotting

Thylakoid isolation was essentially done as described in [20]. Thylakoid proteins were separated by SDS-PAGE using 15% (w/v) acrylamide gels with 6 M urea. Blue native PAGE was performed as in [22] with solubilization of the thylakoid membranes using 0.75% (w/v) *n*-dodecyl-β-D-maltoside. To separate proteins in the second dimension, single lanes were cut out and incubated with 5% β-mercaptoethanol in SDS sample buffer for 30 min at room temperature and then subjected to SDS-PAGE using 15% acrylamide gels. For immunoblotting the gels were incubated in blotting buffer (39 mM glycine, 48 mM Tris, 0.0375% SDS, 20% MetOH) for 30 min before the proteins were transferred to a PVDF membrane (Immobilone, Millipore). The D1, Lhcb1, PsbS and anti-phosphothreonine antibodies were described previously [20], [22], the antibody against CP29 was purchased from Agrisera, Sweden. The ProQ Diamond stain and Sypro Ruby total protein stains were used as in [21].

### Protein characterization by mass spectrometry

Isolated thylakoids were resuspended in 25 mM NH_4_HCO_3_, 10 mM NaF to a final concentration of 2.5 mg of chlorophyll/ml and incubated for 3 h at 22°C with a sequencing grade-modified trypsin from Promega (Madison, WI, USA) at 5 µg of enzyme/mg of chlorophyll and the released peptides were prepared and analyzed as in [21]. The level of phosphorylation for the PSII core proteins was calculated from the ratios of phosphorylated to non-phosphorylated peptide intensities in each LC-MS chromatogram using normalization with the earlier determined flyability ratios for peptide/phosphopeptide pairs from the D1, D2, CP43 and PbsH proteins [21]. For relative quantitative MS studies the released peptides from wild-type and *stn7* mutant thylakoids were esterified using either d0-methyl alcohol or d3-methyl d-alcohol (Sigma Aldrich). The isotope-labeled peptides were mixed 1∶1 before phosphopeptide enrichment using IMAC [24]. For thylakoid protein complex identification isolated thylakoid membranes from wild type plants exposed to normal or high light for 3 hours were separated using blue native PAGE; the bands corresponding to PSII supercomplexes, PSII dimers, PSII monomers and LHCII trimers were excised from the gel and treated with trypsin (sequencing grade modified trypsin, Promega, Madison, WI, USA) essentially according to the described procedure [50]. Peptides were analyzed using an on-line nano-flow HPLC system (EASY-nLC; Proxeon, Bruker Daltonics) in conjugation with the mass spectrometer HCTultra PTM Discovery System (Bruker Daltonics). A 20 mm×100 µm pre column followed by a 100 mm×75 µm analytical column both packed with reverse-phase C18 were used for separation at a flow rate of 300 nL/min. The gradient buffers used were 0.1% formic acid in water (A) and 0.1% formic acid in 100% acetonitrile (B). Separation was performed for 240 min as follow: 0–15% B in first 110 min; 15%–40% B in 110–200 min; 40%–100% B in 200–220 min and 100% B in 220–240 min. The automated online tandem MS analyses were performed using alternating collision induced dissociation and electron transfer dissociation of peptide ions.

## Supporting Information

Figure S1 Peptide identification views from MASCOT MS data analyses of phosphorylated peptides sequenced by collision induced dissociation (CID) or electron transfer dissociation (ETD) of their ions in the samples from the high-light-treated plants. The spectra and corresponding lists of singly and doubly charged fragment ions identified in the MASCOT search are shown.
(DOC)

Figure S2 The sums of the tandem mass spectra for CP29 isoforms Lhcb4.1 and Lhcb4.2 and for two core proteins from each of the three complexes: PSII (D1 and D2 proteins), PSI (PsaA and PsaB proteins) and LHCII (Lhcb1 and Lhcb2 proteins) counted in the gel bands corresponding to PSII supercomplex, PSII dimer, PSII monomer and LHCII trimer from plants exposed for three hours to either normal or high light.
(DOC)

Figure S3 Blue native gel separation and analyses of the thylakoid membrane complexes from the *stn7*, *stn8* and *stn7stn8* mutant plants exposed for three hours to either normal or high light, as indicated. Immunoblotting was done using antibodies against the D1, Lhcb1 and PsbS proteins, as indicated.
(EPS)
