# Supplementary material for: High Light Induced Disassembly of Photosystem II Supercomplexes in Arabidopsis Requires STN7-Dependent Phosphorylation of CP29
Source: PLoS One. 2011 Sep 7;6(9):e24565. doi: 10.1371/journal.pone.0024565 (PMC3168523; doi:10.1371/journal.pone.0024565)
Supplement: Figure S1 — Peptide identification views from MASCOT MS data analyses of phosphorylated peptides sequenced by collision induced dissociation (CID) or electron transfer dissociation (ETD) of their ions in the samples from the high-light-treated plants. The spectra and corresponding lists of singly and doubly charged fragment ions identified in the MASCOT search are shown. (DOC) [file pone.0024565.s001.doc]

ETD / MS/MS Fragmentation of TDSSAAAAAAPATK

Found in gi|15235503, PSAD-1 (photosystem I subunit D-1) [Arabidopsis thaliana]


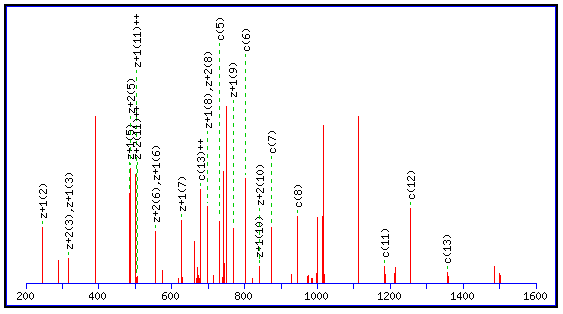


T1 : Phospho (ST), with neutral losses 0.0000(shown in table), 97.9769

S3 : Phospho (ST), with neutral losses 0.0000(shown in table), 97.9769

S4 : Phospho (ST), with neutral losses 0.0000(shown in table), 97.9769

Matches : 23/148 fragment ions using 45 most intense peaks

Ions Score: 37 Expect: 0.0023

| **#** | **c** | **c^++^** | **Seq.** | **y** | **y^++^** | **z+1** | **z+1^++^** | **z+2** | **z+2^++^** | **#** |
| --- | --- | --- | --- | --- | --- | --- | --- | --- | --- | --- |
| **1** | 199.0478 | 100.0276 | **T** |  |  |  |  |  |  | **14** |
| **2** | 328.0904 | 164.5489 | **D** | 1319.5280 | 660.2676 | 1303.5093 | 652.2583 | 1304.5171 | 652.7622 | **13** |
| **3** | 495.0888 | 248.0480 | **S** | 1190.4854 | 595.7463 | 1174.4667 | 587.7370 | 1175.4745 | 588.2409 | **12** |
| **4** | 662.0871 | 331.5472 | **S** | 1023.4871 | 512.2472 | 1007.4683 | **504.2378** | 1008.4762 | **504.7417** | **11** |
| **5** | ***733.1243*** | 367.0658 | **A** | 856.4887 | 428.7480 | ***840.4700*** | 420.7386 | **841.4778** | 421.2425 | **10** |
| **6** | ***804.1614*** | 402.5843 | **A** | 785.4516 | 393.2294 | ***769.4329*** | 385.2201 | 770.4407 | 385.7240 | **9** |
| **7** | ***875.1985*** | 438.1029 | **A** | 714.4145 | 357.7109 | ***698.3957*** | 349.7015 | **699.4036** | 350.2054 | **8** |
| **8** | ***946.2356*** | 473.6214 | **A** | 643.3774 | 322.1923 | ***627.3586*** | 314.1830 | 628.3665 | 314.6869 | **7** |
| **9** | **1017.2727** | 509.1400 | **A** | 572.3402 | 286.6738 | ***556.3215*** | 278.6644 | **557.3293** | 279.1683 | **6** |
| **10** | **1088.3098** | 544.6586 | **A** | 501.3031 | 251.1552 | ***485.2844*** | 243.1458 | **486.2922** | 243.6498 | **5** |
| **11** | ***1185.3626*** | 593.1849 | **P** | 430.2660 | 215.6366 | **414.2473** | 207.6273 | 415.2551 | 208.1312 | **4** |
| **12** | ***1256.3997*** | 628.7035 | **A** | 333.2132 | 167.1103 | ***317.1945*** | 159.1009 | **318.2023** | 159.6048 | **3** |
| **13** | ***1357.4474*** | 679.2273 | **T** | 262.1761 | 131.5917 | ***246.1574*** | 123.5823 | 247.1652 | 124.0863 | **2** |
| **14** |  |  | **K** | 161.1285 | 81.0679 | 145.1097 | 73.0585 | 146.1176 | 73.5624 | **1** |

CID / MS/MS Fragmentation of NLAGDVIGTRTEAADAK

Found in gi|13877547, chlorophyll a/b-binding protein CP29 [Arabidopsis thaliana]


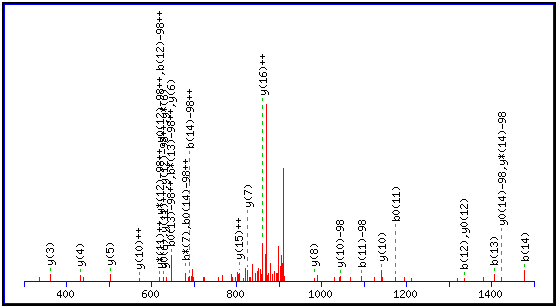


T11 : Phospho (ST), with neutral losses 97.9769 (shown in table),

Matches : 32/276 fragment ions using 39 most intense peaks

Ions Score: 57 Expect: 0.0036


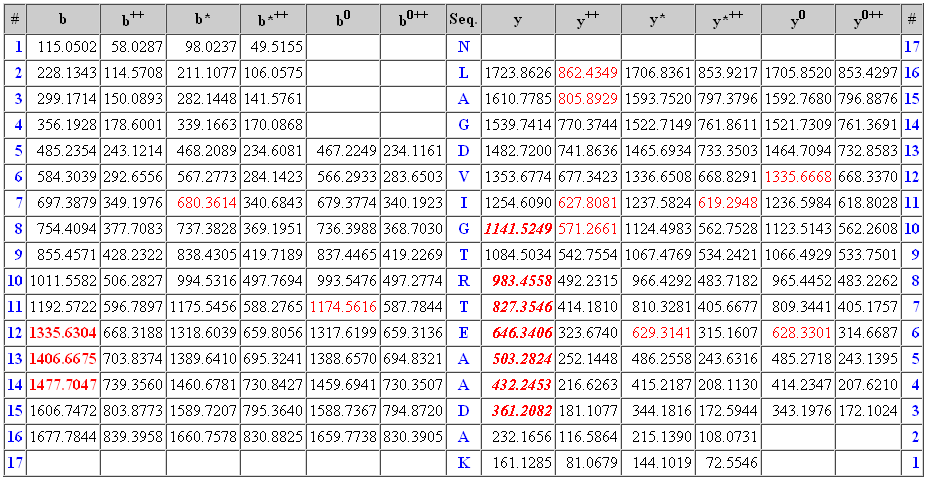


ETD / MS/MS Fragmentation of NLAGDVIGTRTEAADAK

Found in gi|13877547, chlorophyll a/b-binding protein CP29 [Arabidopsis thaliana]


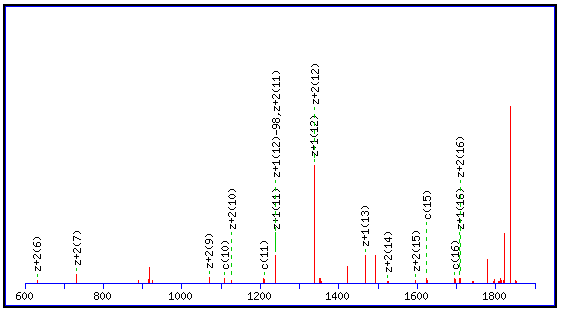


T9 : Phospho (ST), with neutral losses 0.0000(shown in table), 97.9769

Matches : 18/192 fragment ions using 27 most intense peaks

Ions Score: 63 Expect: 0.00083

| **#** | **c** | **c^++^** | **Seq.** | **y** | **y^++^** | **z+1** | **z+1^++^** | **z+2** | **z+2^++^** | **#** |
| --- | --- | --- | --- | --- | --- | --- | --- | --- | --- | --- |
| **1** | 132.0768 | 66.5420 | **N** |  |  |  |  |  |  | **17** |
| **2** | 245.1608 | 123.0840 | **L** | 1723.8626 | 862.4349 | **1707.8439** | 854.4256 | ***1708.8517*** | 854.9295 | **16** |
| **3** | 316.1979 | 158.6026 | **A** | 1610.7785 | 805.8929 | 1594.7598 | 797.8835 | ***1595.7676*** | 798.3875 | **15** |
| **4** | 373.2194 | 187.1133 | **G** | 1539.7414 | 770.3744 | 1523.7227 | 762.3650 | ***1524.7305*** | 762.8689 | **14** |
| **5** | 502.2620 | 251.6346 | **D** | 1482.7200 | 741.8636 | **1466.7012** | 733.8543 | 1467.7091 | 734.3582 | **13** |
| **6** | 601.3304 | 301.1688 | **V** | 1353.6774 | 677.3423 | **1337.6586** | 669.3330 | ***1338.6665*** | 669.8369 | **12** |
| **7** | 714.4145 | 357.7109 | **I** | 1254.6090 | 627.8081 | **1238.5902** | 619.7988 | ***1239.5981*** | 620.3027 | **11** |
| **8** | 771.4359 | 386.2216 | **G** | 1141.5249 | 571.2661 | 1125.5062 | 563.2567 | ***1126.5140*** | 563.7606 | **10** |
| **9** | 952.4499 | 476.7286 | **T** | 1084.5034 | 542.7554 | 1068.4847 | 534.7460 | ***1069.4925*** | 535.2499 | **9** |
| **10** | **1108.5511** | 554.7792 | **R** | 903.4894 | 452.2483 | 887.4707 | 444.2390 | 888.4785 | 444.7429 | **8** |
| **11** | **1209.5987** | 605.3030 | **T** | 747.3883 | 374.1978 | 731.3696 | 366.1884 | ***732.3774*** | 366.6923 | **7** |
| **12** | 1352.6570 | 676.8321 | **E** | 646.3406 | 323.6740 | 630.3219 | 315.6646 | ***631.3297*** | 316.1685 | **6** |
| **13** | 1423.6941 | 712.3507 | **A** | 503.2824 | 252.1448 | 487.2637 | 244.1355 | 488.2715 | 244.6394 | **5** |
| **14** | 1494.7312 | 747.8692 | **A** | 432.2453 | 216.6263 | 416.2266 | 208.6169 | 417.2344 | 209.1208 | **4** |
| **15** | **1623.7738** | 812.3905 | **D** | 361.2082 | 181.1077 | 345.1894 | 173.0984 | 346.1973 | 173.6023 | **3** |
| **16** | **1694.8109** | 847.9091 | **A** | 232.1656 | 116.5864 | 216.1468 | 108.5771 | 217.1547 | 109.0810 | **2** |
| **17** |  |  | **K** | 161.1285 | 81.0679 | 145.1097 | 73.0585 | 146.1176 | 73.5624 | **1** |

ETD / MS/MS Fragmentation of NLYGEVIGTRTEAVDPK, LHCB4.2 (light harvesting complex PSII); chlorophyll binding [Arabidopsis thaliana]


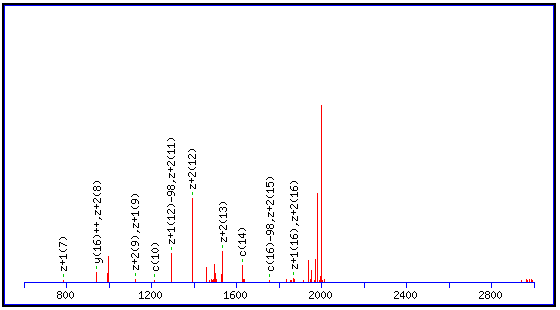


T11 : Phospho (ST), with neutral losses 0.0000(shown in table), 97.9769

Matches : 15/192 fragment ions using 13 most intense peaks

Ions Score: 56 Expect: 0.0042

| **#** | **c** | **c^++^** | **Seq.** | **y** | **y^++^** | **z+1** | **z+1^++^** | **z+2** | **z+2^++^** | **#** |
| --- | --- | --- | --- | --- | --- | --- | --- | --- | --- | --- |
| **1** | 132.0768 | 66.5420 | **N** |  |  |  |  |  |  | **17** |
| **2** | 245.1608 | 123.0840 | **L** | 1883.9514 | **942.4794** | **1867.9327** | 934.4700 | **1868.9405** | 934.9739 | **16** |
| **3** | 408.2241 | 204.6157 | **Y** | 1770.8674 | 885.9373 | 1754.8486 | 877.9280 | **1755.8565** | 878.4319 | **15** |
| **4** | 465.2456 | 233.1264 | **G** | 1607.8040 | 804.4057 | 1591.7853 | 796.3963 | 1592.7931 | 796.9002 | **14** |
| **5** | 608.3039 | 304.6556 | **E** | 1550.7826 | 775.8949 | 1534.7638 | 767.8856 | **1535.7717** | 768.3895 | **13** |
| **6** | 707.3723 | 354.1898 | **V** | 1407.7243 | 704.3658 | 1391.7056 | 696.3564 | **1392.7134** | 696.8604 | **12** |
| **7** | 820.4563 | 410.7318 | **I** | 1308.6559 | 654.8316 | 1292.6372 | 646.8222 | **1293.6450** | 647.3261 | **11** |
| **8** | 877.4778 | 439.2425 | **G** | 1195.5718 | 598.2896 | 1179.5531 | 590.2802 | 1180.5609 | 590.7841 | **10** |
| **9** | 1058.4918 | 529.7495 | **T** | 1138.5504 | 569.7788 | **1122.5317** | 561.7695 | **1123.5395** | 562.2734 | **9** |
| **10** | **1214.5929** | 607.8001 | **R** | 957.5364 | 479.2718 | 941.5176 | 471.2625 | **942.5255** | 471.7664 | **8** |
| **11** | 1315.6406 | 658.3239 | **T** | 801.4353 | 401.2213 | **785.4165** | 393.2119 | 786.4244 | 393.7158 | **7** |
| **12** | 1458.6988 | 729.8531 | **E** | 700.3876 | 350.6974 | 684.3689 | 342.6881 | 685.3767 | 343.1920 | **6** |
| **13** | 1529.7360 | 765.3716 | **A** | 557.3293 | 279.1683 | 541.3106 | 271.1589 | 542.3184 | 271.6629 | **5** |
| **14** | **1628.8044** | 814.9058 | **V** | 486.2922 | 243.6498 | 470.2735 | 235.6404 | 471.2813 | 236.1443 | **4** |
| **15** | 1757.8470 | 879.4271 | **D** | 387.2238 | 194.1155 | 371.2051 | 186.1062 | 372.2129 | 186.6101 | **3** |
| **16** | 1854.8997 | 927.9535 | **P** | 258.1812 | 129.5942 | 242.1625 | 121.5849 | 243.1703 | 122.0888 | **2** |
| **17** |  |  | **K** | 161.1285 | 81.0679 | 145.1097 | 73.0585 | 146.1176 | 73.5624 | **1** |

CID / MS/MS Fragmentation of NLYGEVIGTRTEAVDPK

Found in gi|15231990, LHCB4.2 (light harvesting complex PSII); chlorophyll binding [Arabidopsis thaliana]


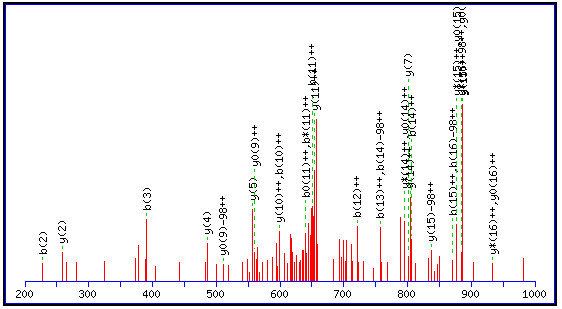


T9 : Phospho (ST), with neutral losses 97.9769 (shown in table),

Matches : 31/276 fragment ions using 50 most intense peaks

Ions Score: 65 Expect: 0.0093

| **#** | **b** | **b^++^** | **b*** | **b*^++^** | **b^0^** | **b^0++^** | **Seq.** | **y** | **y^++^** | **y*** | **y*^++^** | **y^0^** | **y^0++^** | **#** |
| --- | --- | --- | --- | --- | --- | --- | --- | --- | --- | --- | --- | --- | --- | --- |
| **1** | 115.0502 | 58.0287 | 98.0237 | 49.5155 |  |  | **N** |  |  |  |  |  |  | **17** |
| **2** | **228.1343** | 114.5708 | 211.1077 | 106.0575 |  |  | **L** | 1883.9514 | 942.4794 | 1866.9249 | **933.9661** | 1865.9409 | **933.4741** | **16** |
| **3** | **391.1976** | 196.1024 | 374.1710 | 187.5892 |  |  | **Y** | 1770.8674 | **885.9373** | 1753.8408 | **877.4240** | 1752.8568 | **876.9320** | **15** |
| **4** | 448.2191 | 224.6132 | 431.1925 | 216.0999 |  |  | **G** | 1607.8040 | **804.4057** | 1590.7775 | **795.8924** | 1589.7935 | **795.4004** | **14** |
| **5** | 591.2773 | 296.1423 | 574.2508 | 287.6290 | 573.2667 | 287.1370 | **E** | 1550.7826 | 775.8949 | 1533.7560 | 767.3816 | 1532.7720 | 766.8896 | **13** |
| **6** | 690.3457 | 345.6765 | 673.3192 | 337.1632 | 672.3352 | 336.6712 | **V** | 1407.7243 | 704.3658 | 1390.6978 | 695.8525 | 1389.7138 | 695.3605 | **12** |
| **7** | 803.4298 | 402.2185 | 786.4032 | 393.7053 | 785.4192 | 393.2132 | **I** | 1308.6559 | **654.8316** | 1291.6294 | 646.3183 | 1290.6453 | 645.8263 | **11** |
| **8** | 860.4512 | 430.7293 | 843.4247 | 422.2160 | 842.4407 | 421.7240 | **G** | 1195.5718 | **598.2896** | 1178.5453 | 589.7763 | 1177.5613 | 589.2843 | **10** |
| **9** | 1041.4653 | 521.2363 | 1024.4387 | 512.7230 | 1023.4547 | 512.2310 | **T** | **1138.5504** | 569.7788 | 1121.5238 | 561.2656 | 1120.5398 | **560.7735** | **9** |
| **10** | 1197.5664 | ***599.2868*** | 1180.5398 | 590.7735 | 1179.5558 | 590.2815 | **R** | 957.5364 | 479.2718 | 940.5098 | 470.7585 | 939.5258 | 470.2665 | **8** |
| **11** | 1298.6140 | ***649.8107*** | 1281.5875 | **641.2974** | 1280.6035 | **640.8054** | **T** | **801.4353** | 401.2213 | 784.4087 | 392.7080 | 783.4247 | 392.2160 | **7** |
| **12** | 1441.6723 | ***721.3398*** | 1424.6457 | 712.8265 | 1423.6617 | 712.3345 | **E** | **700.3876** | 350.6974 | 683.3610 | 342.1842 | 682.3770 | 341.6921 | **6** |
| **13** | 1512.7094 | ***756.8583*** | 1495.6829 | 748.3451 | 1494.6988 | 747.8531 | **A** | **557.3293** | 279.1683 | 540.3028 | 270.6550 | 539.3188 | 270.1630 | **5** |
| **14** | 1611.7778 | ***806.3925*** | 1594.7513 | 797.8793 | 1593.7673 | 797.3873 | **V** | **486.2922** | 243.6498 | 469.2657 | 235.1365 | 468.2817 | 234.6445 | **4** |
| **15** | 1740.8204 | ***870.9138*** | 1723.7939 | 862.4006 | 1722.8098 | 861.9086 | **D** | 387.2238 | 194.1155 | 370.1973 | 185.6023 | 369.2132 | 185.1103 | **3** |
| **16** | 1837.8732 | 919.4402 | 1820.8466 | 910.9270 | 1819.8626 | 910.4349 | **P** | **258.1812** | 129.5942 | 241.1547 | 121.0810 |  |  | **2** |
| **17** |  |  |  |  |  |  | **K** | 161.1285 | 81.0679 | 144.1019 | 72.5546 |  |  | **1** |

CID / MS/MS Fragmentation of FGFGTKK

Found in LHCB4.2 (light harvesting complex PSII); chlorophyll binding [Arabidopsis thaliana]


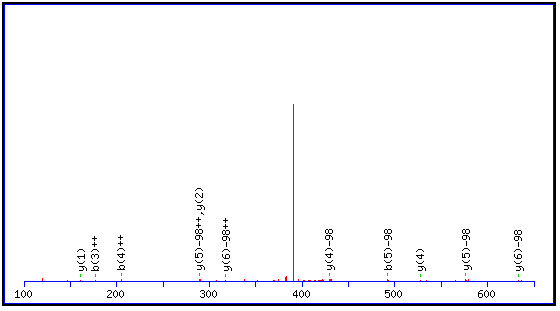


T5 : Phospho (ST), with neutral losses 97.9769(shown in table), 0.0000

Matches : 12/84 fragment ions using 27 most intense peaks

Ions Score: 41 Expect: 1.2e-05

| **#** | **b** | **b^++^** | **b*** | **b*^++^** | **b^0^** | **b^0++^** | **Seq.** | **y** | **y^++^** | **y*** | **y*^++^** | **y^0^** | **y^0++^** | **#** |
| --- | --- | --- | --- | --- | --- | --- | --- | --- | --- | --- | --- | --- | --- | --- |
| **1** | 148.0757 | 74.5415 |  |  |  |  | **F** |  |  |  |  |  |  | **7** |
| **2** | **205.0972** | 103.0522 |  |  |  |  | **G** | **633.3719** | **317.1896** | 616.3453 | 308.6763 | 615.3613 | 308.1843 | **6** |
| **3** | 352.1656 | **176.5864** |  |  |  |  | **F** | **576.3504** | **288.6788** | 559.3239 | 280.1656 | 558.3398 | 279.6736 | **5** |
| **4** | 409.1870 | **205.0972** |  |  |  |  | **G** | ***429.2820*** | 215.1446 | 412.2554 | 206.6314 | 411.2714 | 206.1394 | **4** |
| **5** | **492.2241** | 246.6157 |  |  | 474.2136 | 237.6104 | **T** | **372.2605** | 186.6339 | 355.2340 | 178.1206 | 354.2500 | 177.6286 | **3** |
| **6** | 620.3191 | 310.6632 | 603.2926 | 302.1499 | 602.3085 | 301.6579 | **K** | ***289.2234*** | 145.1153 | 272.1969 | 136.6021 |  |  | **2** |
| **7** |  |  |  |  |  |  | **K** | ***161.1285*** | 81.0679 | 144.1019 | 72.5546 |  |  | **1** |

CID / MS/MS Fragmentation of TLFNGTLALAGR

Found in Photosystem II CP43 chlorophyll protein [Arabidopsis thaliana]


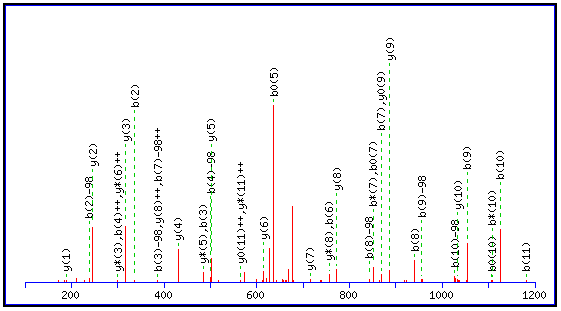


T1 : Phospho (ST), with neutral losses 0.0000(shown in table), 97.9769Matches : 9/76

N-term : Acetyl (N-term)

fragment ions using 38 most intense peaks

Ions Score: 79 Expect: 0.00044

| **#** | **b** | **b^++^** | **b*** | **b*^++^** | **b^0^** | **b^0++^** | **Seq.** | **y** | **y^++^** | **y*** | **y*^++^** | **y^0^** | **y^0++^** | **#** |
| --- | --- | --- | --- | --- | --- | --- | --- | --- | --- | --- | --- | --- | --- | --- |
| **1** | 224.0319 | 112.5196 |  |  | 206.0213 | 103.5143 | **T** |  |  |  |  |  |  | **12** |
| **2** | **337.1159** | 169.0616 |  |  | 319.1053 | 160.0563 | **L** | 1146.6630 | 573.8351 | 1129.6364 | 565.3218 | 1128.6524 | 564.8298 | **11** |
| **3** | **484.1843** | 242.5958 |  |  | 466.1738 | 233.5905 | **F** | **1033.5789** | 517.2931 | 1016.5524 | 508.7798 | 1015.5683 | 508.2878 | **10** |
| **4** | 598.2273 | 299.6173 | 581.2007 | 291.1040 | 580.2167 | 290.6120 | **N** | **886.5105** | 443.7589 | 869.4839 | 435.2456 | 868.4999 | 434.7536 | **9** |
| **5** | 655.2487 | 328.1280 | 638.2222 | 319.6147 | 637.2382 | 319.1227 | **G** | **772.4676** | 386.7374 | 755.4410 | 378.2241 | 754.4570 | 377.7321 | **8** |
| **6** | **756.2964** | 378.6518 | 739.2698 | 370.1386 | 738.2858 | 369.6466 | **T** | **715.4461** | 358.2267 | 698.4196 | 349.7134 | 697.4355 | 349.2214 | **7** |
| **7** | **869.3805** | 435.1939 | 852.3539 | 426.6806 | 851.3699 | 426.1886 | **L** | **614.3984** | 307.7028 | 597.3719 | 299.1896 |  |  | **6** |
| **8** | **940.4176** | 470.7124 | 923.3910 | 462.1992 | 922.4070 | 461.7071 | **A** | **501.3144** | 251.1608 | 484.2878 | 242.6475 |  |  | **5** |
| **9** | **1053.5016** | 527.2545 | 1036.4751 | 518.7412 | 1035.4911 | 518.2492 | **L** | **430.2772** | 215.6423 | 413.2507 | 207.1290 |  |  | **4** |
| **10** | **1124.5388** | 562.7730 | 1107.5122 | 554.2597 | 1106.5282 | 553.7677 | **A** | **317.1932** | 159.1002 | 300.1666 | 150.5870 |  |  | **3** |
| **11** | **1181.5602** | 591.2837 | 1164.5337 | 582.7705 | 1163.5497 | 582.2785 | **G** | **246.1561** | 123.5817 | 229.1295 | 115.0684 |  |  | **2** |
| **12** |  |  |  |  |  |  | **R** | ***189.1346*** | 95.0709 | 172.1081 | 86.5577 |  |  | **1** |

CID / MS/MS Fragmentation of ATTEVGEAPATTTEAETTELPEIVK

Found in PSI-P (PHOTOSYSTEM I P SUBUNIT) [Arabidopsis thaliana]


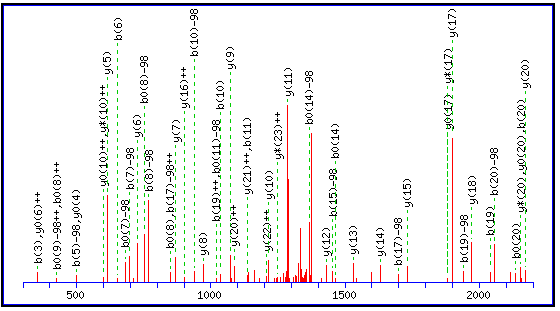


T2 : Phospho (ST), with neutral losses 97.9769(shown in table)

fragment ions using 61 most intense peaks

Ions Score: 96 Expect: 4.2e-07

| **#** | **b** | **b^++^** | **b^0^** | **b^0++^** | **Seq.** | **y** | **y^++^** | **y*** | **y*^++^** | **y^0^** | **y^0++^** | **#** |
| --- | --- | --- | --- | --- | --- | --- | --- | --- | --- | --- | --- | --- |
| **1** | 72.0444 | 36.5258 |  |  | **A** |  |  |  |  |  |  | **25** |
| **2** | 155.0815 | 78.0444 | 137.0709 | 69.0391 | **T** | 2597.3444 | 1299.1758 | 2580.3178 | 1290.6626 | 2579.3338 | 1290.1706 | **24** |
| **3** | 256.1292 | 128.5682 | 238.1186 | 119.5629 | **T** | 2514.3073 | 1257.6573 | 2497.2807 | 1249.1440 | 2496.2967 | 1248.6520 | **23** |
| **4** | 399.1874 | 200.0974 | 381.1769 | 191.0921 | **E** | 2413.2596 | 1207.1334 | 2396.2330 | 1198.6202 | 2395.2490 | 1198.1282 | **22** |
| **5** | **498.2558** | 249.6316 | 480.2453 | 240.6263 | **V** | 2270.2014 | 1135.6043 | 2253.1748 | 1127.0910 | 2252.1908 | 1126.5990 | **21** |
| **6** | 555.2773 | 278.1423 | 537.2667 | 269.1370 | **G** | ***2171.1329*** | 1086.0701 | 2154.1064 | 1077.5568 | 2153.1224 | 1077.0648 | **20** |
| **7** | **698.3355** | 349.6714 | 680.3250 | 340.6661 | **E** | 2114.1115 | 1057.5594 | 2097.0849 | 1049.0461 | 2096.1009 | 1048.5541 | **19** |
| **8** | **769.3727** | 385.1900 | 751.3621 | 376.1847 | **A** | ***1971.0532*** | 986.0303 | 1954.0267 | 977.5170 | 1953.0427 | 977.0250 | **18** |
| **9** | 866.4254 | 433.7164 | 848.4149 | 424.7111 | **P** | ***1900.0161*** | 950.5117 | 1882.9896 | 941.9984 | 1882.0056 | 941.5064 | **17** |
| **10** | **937.4625** | 469.2349 | 919.4520 | 460.2296 | **A** | 1802.9634 | 901.9853 | 1785.9368 | 893.4720 | 1784.9528 | 892.9800 | **16** |
| **11** | 1038.5102 | 519.7587 | 1020.4997 | 510.7535 | **T** | **1731.9262** | 866.4668 | 1714.8997 | 857.9535 | 1713.9157 | 857.4615 | **15** |
| **12** | 1139.5579 | 570.2826 | 1121.5473 | 561.2773 | **T** | **1630.8786** | 815.9429 | 1613.8520 | 807.4296 | 1612.8680 | 806.9376 | **14** |
| **13** | 1240.6056 | 620.8064 | 1222.5950 | 611.8011 | **T** | **1529.8309** | 765.4191 | 1512.8043 | 756.9058 | 1511.8203 | 756.4138 | **13** |
| **14** | 1383.6638 | 692.3355 | 1365.6533 | 683.3303 | **E** | **1428.7832** | 714.8952 | 1411.7567 | 706.3820 | 1410.7726 | 705.8900 | **12** |
| **15** | **1454.7009** | 727.8541 | 1436.6904 | 718.8488 | **A** | **1285.7250** | 643.3661 | 1268.6984 | 634.8528 | 1267.7144 | 634.3608 | **11** |
| **16** | 1597.7592 | 799.3832 | 1579.7486 | 790.3779 | **E** | **1214.6878** | 607.8476 | 1197.6613 | 599.3343 | 1196.6773 | 598.8423 | **10** |
| **17** | **1698.8069** | 849.9071 | 1680.7963 | 840.9018 | **T** | **1071.6296** | 536.3184 | 1054.6031 | 527.8052 | 1053.6190 | 527.3132 | **9** |
| **18** | 1799.8545 | 900.4309 | 1781.8440 | 891.4256 | **T** | **970.5819** | 485.7946 | 953.5554 | 477.2813 | 952.5714 | 476.7893 | **8** |
| **19** | **1942.9128** | 971.9600 | 1924.9022 | 962.9547 | **E** | **869.5342** | 435.2708 | 852.5077 | 426.7575 | 851.5237 | 426.2655 | **7** |
| **20** | **2055.9968** | 1028.5021 | 2037.9863 | 1019.4968 | **L** | **726.4760** | 363.7416 | 709.4495 | 355.2284 | 708.4654 | 354.7364 | **6** |
| **21** | 2153.0496 | 1077.0284 | 2135.0390 | 1068.0232 | **P** | ***613.3919*** | 307.1996 | 596.3654 | 298.6863 | 595.3814 | 298.1943 | **5** |
| **22** | 2296.1078 | 1148.5576 | 2278.0973 | 1139.5523 | **E** | 516.3392 | 258.6732 | 499.3126 | 250.1600 | 498.3286 | 249.6679 | **4** |
| **23** | 2409.1919 | 1205.0996 | 2391.1813 | 1196.0943 | **I** | 373.2809 | 187.1441 | 356.2544 | 178.6308 |  |  | **3** |
| **24** | 2508.2603 | 1254.6338 | 2490.2498 | 1245.6285 | **V** | 260.1969 | 130.6021 | 243.1703 | 122.0888 |  |  | **2** |
| **25** |  |  |  |  | **K** | 161.1285 | 81.0679 | 144.1019 | 72.5546 |  |  | **1** |

CID / MS/MS Fragmentation of ATTEVGEAPATTTEAETTELPEIVK

Found in PSI-P (PHOTOSYSTEM I P SUBUNIT) [Arabidopsis thaliana]


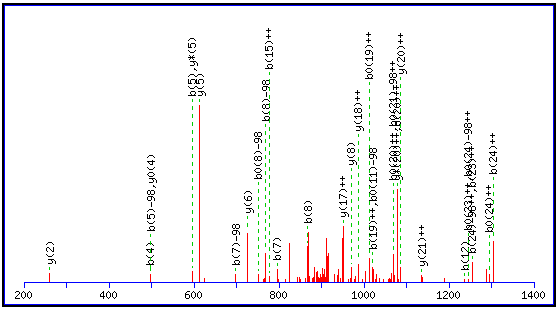


T3 : Phospho (ST), with neutral losses 0.0000(shown in table), 97.9769

fragment ions using 40 most intense peaks

Ions Score: 46 Expect: 0.0088

| **#** | **b** | **b^++^** | **b^0^** | **b^0++^** | **Seq.** | **y** | **y^++^** | **y*** | **y*^++^** | **y^0^** | **y^0++^** | **#** |
| --- | --- | --- | --- | --- | --- | --- | --- | --- | --- | --- | --- | --- |
| **1** | 72.0444 | 36.5258 |  |  | **A** |  |  |  |  |  |  | **25** |
| **2** | 173.0921 | 87.0497 | 155.0815 | 78.0444 | **T** | 2695.3213 | 1348.1643 | 2678.2947 | 1339.6510 | 2677.3107 | 1339.1590 | **24** |
| **3** | 354.1061 | 177.5567 | 336.0955 | 168.5514 | **T** | 2594.2736 | 1297.6404 | 2577.2471 | 1289.1272 | 2576.2630 | 1288.6352 | **23** |
| **4** | **497.1643** | 249.0858 | 479.1538 | 240.0805 | **E** | 2413.2596 | 1207.1334 | 2396.2330 | 1198.6202 | 2395.2490 | 1198.1282 | **22** |
| **5** | **596.2327** | 298.6200 | 578.2222 | 289.6147 | **V** | 2270.2014 | **1135.6043** | 2253.1748 | 1127.0910 | 2252.1908 | 1126.5990 | **21** |
| **6** | **653.2542** | 327.1307 | 635.2436 | 318.1255 | **G** | 2171.1329 | **1086.0701** | 2154.1064 | 1077.5568 | 2153.1224 | 1077.0648 | **20** |
| **7** | **796.3124** | 398.6599 | 778.3019 | 389.6546 | **E** | 2114.1115 | 1057.5594 | 2097.0849 | 1049.0461 | 2096.1009 | 1048.5541 | **19** |
| **8** | **867.3496** | 434.1784 | 849.3390 | 425.1731 | **A** | 1971.0532 | **986.0303** | 1954.0267 | 977.5170 | 1953.0427 | 977.0250 | **18** |
| **9** | 964.4023 | 482.7048 | 946.3918 | 473.6995 | **P** | 1900.0161 | **950.5117** | 1882.9896 | 941.9984 | 1882.0056 | 941.5064 | **17** |
| **10** | 1035.4394 | 518.2234 | 1017.4289 | 509.2181 | **A** | 1802.9634 | 901.9853 | 1785.9368 | 893.4720 | 1784.9528 | 892.9800 | **16** |
| **11** | 1136.4871 | 568.7472 | 1118.4765 | 559.7419 | **T** | 1731.9262 | 866.4668 | 1714.8997 | 857.9535 | 1713.9157 | 857.4615 | **15** |
| **12** | ***1237.5348*** | 619.2710 | 1219.5242 | 610.2658 | **T** | 1630.8786 | 815.9429 | 1613.8520 | 807.4296 | 1612.8680 | 806.9376 | **14** |
| **13** | 1338.5825 | 669.7949 | 1320.5719 | 660.7896 | **T** | 1529.8309 | 765.4191 | 1512.8043 | 756.9058 | 1511.8203 | 756.4138 | **13** |
| **14** | 1481.6407 | 741.3240 | 1463.6301 | 732.3187 | **E** | 1428.7832 | 714.8952 | 1411.7567 | 706.3820 | 1410.7726 | 705.8900 | **12** |
| **15** | 1552.6778 | ***776.8426*** | 1534.6673 | 767.8373 | **A** | 1285.7250 | 643.3661 | 1268.6984 | 634.8528 | 1267.7144 | 634.3608 | **11** |
| **16** | 1695.7361 | 848.3717 | 1677.7255 | 839.3664 | **E** | 1214.6878 | 607.8476 | 1197.6613 | 599.3343 | 1196.6773 | 598.8423 | **10** |
| **17** | 1796.7838 | 898.8955 | 1778.7732 | 889.8902 | **T** | **1071.6296** | 536.3184 | 1054.6031 | 527.8052 | 1053.6190 | 527.3132 | **9** |
| **18** | 1897.8314 | 949.4194 | 1879.8209 | 940.4141 | **T** | **970.5819** | 485.7946 | 953.5554 | 477.2813 | 952.5714 | 476.7893 | **8** |
| **19** | 2040.8897 | ***1020.9485*** | 2022.8791 | 1011.9432 | **E** | **869.5342** | 435.2708 | 852.5077 | 426.7575 | 851.5237 | 426.2655 | **7** |
| **20** | 2153.9737 | ***1077.4905*** | 2135.9632 | 1068.4852 | **L** | **726.4760** | 363.7416 | 709.4495 | 355.2284 | 708.4654 | 354.7364 | **6** |
| **21** | 2251.0265 | 1126.0169 | 2233.0159 | 1117.0116 | **P** | **613.3919** | 307.1996 | 596.3654 | 298.6863 | 595.3814 | 298.1943 | **5** |
| **22** | 2394.0847 | 1197.5460 | 2376.0742 | 1188.5407 | **E** | 516.3392 | 258.6732 | 499.3126 | 250.1600 | 498.3286 | 249.6679 | **4** |
| **23** | 2507.1688 | ***1254.0880*** | 2489.1582 | 1245.0828 | **I** | 373.2809 | 187.1441 | 356.2544 | 178.6308 |  |  | **3** |
| **24** | 2606.2372 | ***1303.6222*** | 2588.2267 | 1294.6170 | **V** | **260.1969** | 130.6021 | 243.1703 | 122.0888 |  |  | **2** |
| **25** |  |  |  |  | **K** | 161.1285 | 81.0679 | 144.1019 | 72.5546 |  |  | **1** |

CID MS/MS Fragmentation of ATQTVEDSSR

Found in gi|7525062, photosystem II protein H [Arabidopsis thaliana]


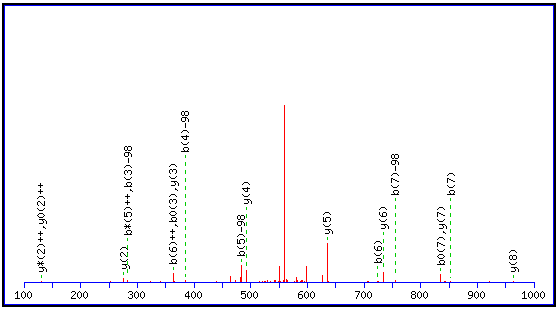


T2 : Phospho (ST), with neutral losses 97.9769(shown in table)

fragment ions using 17 most intense peaks

Ions Score: 64 Expect: 0.00056

| **#** | **b** | **b^++^** | **b*** | **b*^++^** | **b^0^** | **b^0++^** | **Seq.** | **y** | **y^++^** | **y*** | **y*^++^** | **y^0^** | **y^0++^** | **#** |
| --- | --- | --- | --- | --- | --- | --- | --- | --- | --- | --- | --- | --- | --- | --- |
| **1** | 72.0444 | 36.5258 |  |  |  |  | **A** |  |  |  |  |  |  | **10** |
| **2** | 155.0815 | 78.0444 |  |  | 137.0709 | 69.0391 | **T** | 1046.5113 | 523.7593 | 1029.4847 | 515.2460 | 1028.5007 | 514.7540 | **9** |
| **3** | **283.1401** | 142.0737 | 266.1135 | 133.5604 | 265.1295 | 133.0684 | **Q** | **963.4742** | 482.2407 | 946.4476 | 473.7274 | 945.4636 | 473.2354 | **8** |
| **4** | **384.1878** | 192.5975 | 367.1612 | 184.0842 | 366.1772 | 183.5922 | **T** | **835.4156** | 418.2114 | 818.3890 | 409.6982 | 817.4050 | 409.2061 | **7** |
| **5** | **483.2562** | 242.1317 | 466.2296 | 233.6185 | 465.2456 | 233.1264 | **V** | **734.3679** | 367.6876 | 717.3414 | 359.1743 | 716.3573 | 358.6823 | **6** |
| **6** | 626.3144 | 313.6608 | 609.2879 | 305.1476 | 608.3039 | 304.6556 | **E** | **635.2995** | 318.1534 | 618.2729 | 309.6401 | 617.2889 | 309.1481 | **5** |
| **7** | **755.3570** | 378.1821 | 738.3305 | 369.6689 | 737.3464 | 369.1769 | **D** | **492.2413** | 246.6243 | 475.2147 | 238.1110 | 474.2307 | 237.6190 | **4** |
| **8** | 842.3890 | 421.6982 | 825.3625 | 413.1849 | 824.3785 | 412.6929 | **S** | **363.1987** | 182.1030 | 346.1721 | 173.5897 | 345.1881 | 173.0977 | **3** |
| **9** | 929.4211 | 465.2142 | 912.3945 | 456.7009 | 911.4105 | 456.2089 | **S** | **276.1666** | 138.5870 | 259.1401 | 130.0737 | 258.1561 | 129.5817 | **2** |
| **10** |  |  |  |  |  |  | **R** | 189.1346 | 95.0709 | 172.1081 | 86.5577 |  |  | **1** |

ETD MS/MS Fragmentation of ATQTVEDSSR

Found in gi|7525062, photosystem II protein H [Arabidopsis thaliana]


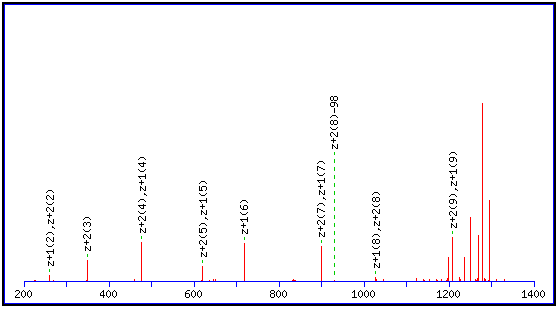


T2 : Phospho (ST), with neutral losses 0.0000(shown in table), 97.9769

T4 : Phospho (ST), with neutral losses 0.0000(shown in table), 97.9769

fragment ions using 14 most intense peaks

Ions Score: 76 Expect: 5.1e-05

| **#** | **c** | **c^++^** | **Seq.** | **y** | **y^++^** | **z+1** | **z+1^++^** | **z+2** | **z+2^++^** | **#** |
| --- | --- | --- | --- | --- | --- | --- | --- | --- | --- | --- |
| **1** | 89.0709 | 45.0391 | **A** |  |  |  |  |  |  | **10** |
| **2** | 270.0849 | 135.5461 | **T** | 1224.4545 | 612.7309 | **1208.4358** | 604.7215 | ***1209.4436*** | 605.2254 | **9** |
| **3** | 398.1435 | 199.5754 | **Q** | 1043.4405 | 522.2239 | **1027.4218** | 514.2145 | ***1028.4296*** | 514.7184 | **8** |
| **4** | 579.1575 | 290.0824 | **T** | 915.3819 | 458.1946 | **899.3632** | 450.1852 | ***900.3710*** | 450.6891 | **7** |
| **5** | 678.2260 | 339.6166 | **V** | 734.3679 | 367.6876 | **718.3492** | 359.6782 | 719.3570 | 360.1821 | **6** |
| **6** | 821.2842 | 411.1457 | **E** | 635.2995 | 318.1534 | **619.2808** | 310.1440 | ***620.2886*** | 310.6479 | **5** |
| **7** | 950.3268 | 475.6670 | **D** | 492.2413 | 246.6243 | **476.2225** | 238.6149 | ***477.2304*** | 239.1188 | **4** |
| **8** | 1037.3588 | 519.1830 | **S** | 363.1987 | 182.1030 | 347.1799 | 174.0936 | ***348.1878*** | 174.5975 | **3** |
| **9** | 1124.3908 | 562.6991 | **S** | 276.1666 | 138.5870 | ***260.1479*** | 130.5776 | ***261.1557*** | 131.0815 | **2** |
| **10** |  |  | **R** | 189.1346 | 95.0709 | 173.1159 | 87.0616 | 174.1237 | 87.5655 | **1** |

CID MS/MS Fragmentation of SGTKFLPSSD

Found in gi|15237201, CaS (Calcium sensing receptor) [Arabidopsis thaliana]


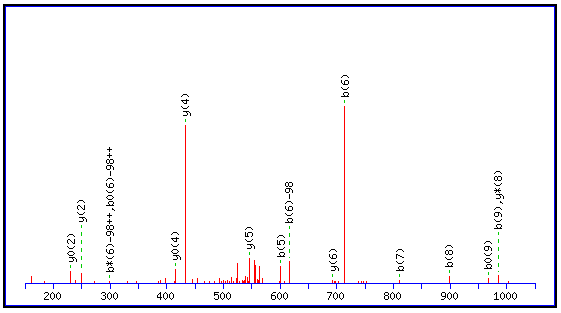


T3 : Phospho (ST), with neutral losses 0.0000(shown in table), 97.9769

fragment ions using 16 most intense peaks

Ions Score: 39 Expect: 0.0015

| **#** | **b** | **b^++^** | **b*** | **b*^++^** | **b^0^** | **b^0++^** | **Seq.** | **y** | **y^++^** | **y*** | **y*^++^** | **y^0^** | **y^0++^** | **#** |
| --- | --- | --- | --- | --- | --- | --- | --- | --- | --- | --- | --- | --- | --- | --- |
| **1** | 88.0393 | 44.5233 |  |  | 70.0287 | 35.5180 | **S** |  |  |  |  |  |  | **10** |
| **2** | 145.0608 | 73.0340 |  |  | 127.0502 | 64.0287 | **G** | 1059.4758 | 530.2415 | 1042.4493 | 521.7283 | 1041.4653 | 521.2363 | **9** |
| **3** | 326.0748 | 163.5410 |  |  | 308.0642 | 154.5357 | **T** | 1002.4544 | 501.7308 | 985.4278 | 493.2175 | 984.4438 | 492.7255 | **8** |
| **4** | 454.1697 | 227.5885 | 437.1432 | 219.0752 | 436.1592 | 218.5832 | **K** | **821.4403** | 411.2238 | 804.4138 | 402.7105 | 803.4298 | 402.2185 | **7** |
| **5** | **601.2382** | 301.1227 | 584.2116 | 292.6094 | 583.2276 | 292.1174 | **F** | **693.3454** | 347.1763 |  |  | 675.3348 | 338.1710 | **6** |
| **6** | **714.3222** | 357.6647 | 697.2957 | 349.1515 | 696.3117 | 348.6595 | **L** | **546.2770** | 273.6421 |  |  | 528.2664 | 264.6368 | **5** |
| **7** | **811.3750** | 406.1911 | 794.3484 | 397.6779 | 793.3644 | 397.1858 | **P** | **433.1929** | 217.1001 |  |  | 415.1823 | 208.0948 | **4** |
| **8** | **898.4070** | 449.7071 | 881.3805 | 441.1939 | 880.3964 | 440.7019 | **S** | **336.1401** | 168.5737 |  |  | 318.1296 | 159.5684 | **3** |
| **9** | **985.4390** | 493.2232 | 968.4125 | 484.7099 | 967.4285 | 484.2179 | **S** | **249.1081** | 125.0577 |  |  | 231.0975 | 116.0524 | **2** |
| **10** |  |  |  |  |  |  | **D** | 162.0761 | 81.5417 |  |  | 144.0655 | 72.5364 | **1** |

CID MS/MS Fragmentation of TIALGK

Found in gi|7525028, photosystem II protein D2 [Arabidopsis thaliana]


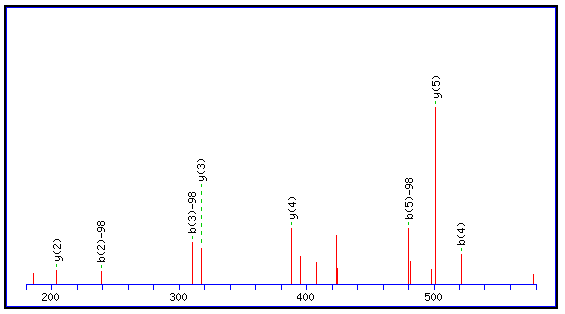


T1 : Phospho (ST), with neutral losses 97.9769(shown in table), 0.0000

N-term : Acetyl (N-term)

fragment ions using 11 most intense peaks

Ions Score: 37 Expect: 0.0041

| **#** | **b** | **b^0^** | **Seq.** | **y** | **y*** | **#** |
| --- | --- | --- | --- | --- | --- | --- |
| **1** | 126.0550 | 108.0444 | **T** |  |  | **6** |
| **2** | **239.1390** | 221.1285 | **I** | **501.3395** | 484.3130 | **5** |
| **3** | **310.1761** | 292.1656 | **A** | **388.2554** | 371.2289 | **4** |
| **4** | **423.2602** | 405.2496 | **L** | **317.2183** | 300.1918 | **3** |
| **5** | **480.2817** | 462.2711 | **G** | **204.1343** | 187.1077 | **2** |
| **6** |  |  | **K** | 147.1128 | 130.0863 | **1** |

CID MS/MS Fragmentation of TAILER

Found in gi|7525013, photosystem II protein D1 [Arabidopsis thaliana]


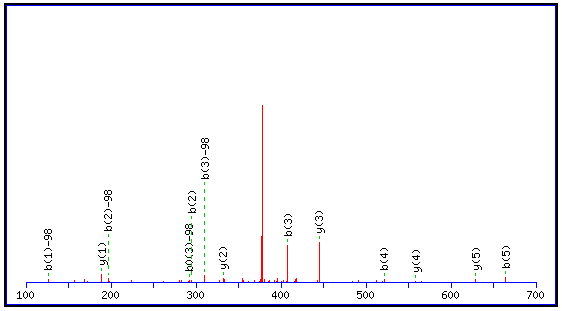


T1 : Phospho (ST), with neutral losses 97.9769(shown in table), 0.0000

N-term : Acetyl (N-term)

fragment ions using 20 most intense peaks

Ions Score: 39 Expect: 0.0024

| **#** | **b** | **b^++^** | **b^0^** | **b^0++^** | **Seq.** | **y** | **y^++^** | **y*** | **y*^++^** | **y^0^** | **y^0++^** | **#** |
| --- | --- | --- | --- | --- | --- | --- | --- | --- | --- | --- | --- | --- |
| **1** | **126.0550** | 63.5311 | 108.0444 | 54.5258 | **T** |  |  |  |  |  |  | **6** |
| **2** | **197.0921** | 99.0497 | 179.0815 | 90.0444 | **A** | **629.3981** | 315.2027 | 612.3715 | 306.6894 | 611.3875 | 306.1974 | **5** |
| **3** | **310.1761** | 155.5917 | 292.1656 | 146.5864 | **I** | **558.3610** | 279.6841 | 541.3344 | 271.1709 | 540.3504 | 270.6788 | **4** |
| **4** | **423.2602** | 212.1337 | 405.2496 | 203.1285 | **L** | **445.2769** | 223.1421 | 428.2504 | 214.6288 | 427.2663 | 214.1368 | **3** |
| **5** | 566.3184 | 283.6629 | 548.3079 | 274.6576 | **E** | **332.1928** | 166.6001 | 315.1663 | 158.0868 | 314.1823 | 157.5948 | **2** |
| **6** |  |  |  |  | **R** | **189.1346** | 95.0709 | 172.1081 | 86.5577 |  |  | **1** |
