# Supplementary material for: High Light Induced Disassembly of Photosystem II Supercomplexes in Arabidopsis Requires STN7-Dependent Phosphorylation of CP29
Source: PLoS One. 2011 Sep 7;6(9):e24565. doi: 10.1371/journal.pone.0024565 (PMC3168523; doi:10.1371/journal.pone.0024565)
Supplement: Figure S2 — The sums of the tandem mass spectra for CP29 isoforms Lhcb4.1 and Lhcb4.2 and for two core proteins from each of the three complexes: PSII (D1 and D2 proteins), PSI (PsaA and PsaB proteins) and LHCII (Lhcb1 and Lhcb2 proteins) counted in the gel bands corresponding to PSII supercomplex, PSII dimer, PSII monomer and LHCII trimer from plants exposed for three hours to either normal or high light. (DOC) [file pone.0024565.s002.doc]

SpectralCounts ± from 4 different BN-Gel- In-Gel-Digestion experiments.

***Normal Light***

*Spectral counts*

**PSII supercomplex**

D1 26 ± 8

D2 33 ± 6

PsaA 26 ± 4

PsaB 32 ± 12

Lhcb1 32 ± 9

Lhcb2 23 ± 3

Lhcb4.1 19 ± 5

Lhcb4.2 27 ± 3

**PSII Dimer**

D1 31 ± 3

D2 36 ± 5

PsaA 22 ± 9

PsaB 28 ± 12

Lhcb1 21 ± 13

Lhcb2 19 ± 7

Lhcb4.1 16 ± 7

Lhcb4.2 22 ± 5

**PSII Monomer**

D1 23 ± 8

D2 16 ± 6

*PsaA n.a.*

*PsaB n.a.*

Lhcb1 23 ± 12

Lhcb2 26 ± 4

Lhcb4.1 10 ± 6

Lhcb4.2 14 ± 4

**LHCII Trimer**

*D1 n.a.*

*D2 n.a.*

*PsaA n.a.*

*PsaB n.a.*

Lhcb1 27 ± 9

Lhcb2 31 ± 11

Lhcb4.1 21 ± 6

Lhcb4.2 27 ± 7

SpectralCounts ± from 4 different BN-Gel- In-Gel-Digestion experiments.

***High Light***

*Spectral counts*

**PSII supercomplex**

D1 13 ± 5

D2 21 ± 7

PsaA 29 ± 9

PsaB 31 ± 13

Lhcb1 20 ± 12

Lhcb2 14 ± 6

Lhcb4.1 10 ± 4

Lhcb4.2 12 ± 4

**PSII Dimer**

D1 35 ± 4

D2 33 ± 8

PsaA 24 ± 6

PsaB 27 ± 16

Lhcb1 18 ± 12

Lhcb2 15 ± 4

Lhcb4.1 25 ± 6

Lhcb4.2 29 ± 8

**PSII Monomer**

D1 19 ± 5

D2 22 ± 5

*PsaA n.a.*

*PsaB n.a.*

Lhcb1 11 ± 14

Lhcb2 13 ± 6

Lhcb4.1 19 ± 5

Lhcb4.2 26 ± 7

**LHCII Trimer**

*D1 n.a.*

*D2 n.a.*

*PsaA n.a.*

*PsaB n.a.*

Lhcb1 38 ± 12

Lhcb2 44 ± 5

Lhcb4.1 26 ± 4

Lhcb4.2 32 ± 7
